# Pre-exposure prophylaxis (PrEP) and medications for opioid use disorder for persons who inject drugs: the CHORUS + randomized controlled trial study protocol

**DOI:** 10.1186/s13722-025-00634-2

**Published:** 2025-12-25

**Authors:** Sarah E. Miller, Kimberly A. Dukes, Carolyn Damato-MacPherson, Christina Psaros, Nancy A. Scott, Jessica L. Taylor, Jordana Muroff, Michael R. Winter, Lisette E. Skiba, Hansel Lugo, Ricardo Cruz, Glorimar Ruiz-Mercado, Natalie D. Crawford, Kenneth H. Mayer, Sabrina A. Assoumou

**Affiliations:** 1 Section of Infectious Diseases, Boston Medical Center, 801 Massachusetts Ave. Crosstown Center, 2nd Floor, Boston, MA 02118 USA; 2 Boston University School of Public Health, Boston, MA USA; 3 Behavioral Medicine Program, Massachusetts General Hospital, Boston, MA USA; 4 Department of Psychiatry, Harvard Medical School, Boston, MA USA; 5 General Internal Medicine, Boston Medical Center, Boston, MA USA; 6 Section of General Internal Medicine, Department of Medicine, Boston University Chobanian and Avedisian School of Medicine, Boston, USA; 7 Boston University School of Social Work, Boston, MA USA; 8 Rollins School of Public Health, Emory University, Atlanta, GA USA; 9 Fenway Health Institute, Boston, MA USA; 10 Section of Infectious Diseases, Department of Medicine, Boston University Chobanian & Avedisian School of Medicine, Boston, MA USA

**Keywords:** Human immunodeficiency virus (HIV), Medications for opioid use disorder (MOUD), Pre-exposure prophylaxis (PrEP), Peer recovery coaching (PRC), Motivational interviewing (MI), HIV self-testing, People who inject opioids (PWIO)

## Abstract

**Background:**

Human immunodeficiency virus (HIV) cases among people who inject drugs increased during the US overdose crisis. Although HIV pre-exposure prophylaxis (PrEP) decreases HIV acquisition, and medications for opioid use disorder (MOUD) reduce overdose deaths, uptake remains suboptimal. The CHORUS + study will test the efficacy of a comprehensive peer recovery coaching intervention to increase PrEP and MOUD initiation and adherence.

**Methods:**

This two-arm RCT will enroll 284 people who inject opioids (PWIO) and are negative for HIV from two sites. Participants randomized to the CHORUS + intervention will receive a study smartphone, motivational interviewing sessions, assistance connecting to PrEP and/or MOUD, and support to access resources addressing social needs such as employment and housing. We will also incorporate adapted ‘Life-Steps for PrEP’ modules to increase adherence. The control arm will receive information on local organizations with access to PrEP and MOUD. Participants will complete assessments at baseline, 1-, 3-, 6-, and 12-month visits. The primary outcome is adherence to HIV PrEP, assessed by tenofovir-diphosphate drug levels at 6-months post enrollment. Secondary outcomes include PrEP adherence assessed at 3- and 12-months, measured by drug levels (3-months), prescription refills, and self-report; and MOUD receipt at 3-, 6-, and 12-months, measured by prescription refills, self-report, and urine toxicology. The primary analysis will employ intent-to-treat logistic regression to assess differences in adherence between treatment arms, adjusting for stratification factors including site, race and sex assigned at birth. We will analyze secondary outcomes using similar methods. We will use multilevel growth curve modeling to evaluate changes in adherence over time by treatment group, incorporating random intercepts and slopes to account for individual trajectories. We will use exploratory multilevel structural equation modeling to assess mediators including HIV risk perception and PrEP/MOUD knowledge to understand pathways that may influence adherence.

**Discussion:**

The CHORUS + intervention integrates a novel, theory-based, peer-delivered, smartphone-supported approach to address HIV prevention and opioid use disorder, while tackling social and structural barriers to care. Findings will inform strategies for linking PWIO to rapid HIV prevention and substance use treatment.

**Trial registration:**

ClinicalTrials.gov number: NCT05769218.

**Supplementary Information:**

The online version contains supplementary material available at 10.1186/s13722-025-00634-2.

## Background

During the ongoing US overdose crisis, human immunodeficiency virus (HIV) incidence has risen among persons who inject drugs (PWID) [1, 2]. Although the overall number of new HIV cases has decreased nationally, the proportion of infections attributed to injection drug use remains consistent [3]. In some states, including Massachusetts, 20% of new HIV infections in 2021 were attributed to injection drug use [4]. The co-occurrence of HIV and opioid use disorder (OUD) form a syndemic, which describes intersecting OUD epidemics that are observed in the same population and leads to worse outcomes than if they had occurred individually [5]. As such, effectively tackling HIV and OUD requires a multi-pronged and comprehensive approach. Previous studies have demonstrated the critical need to concurrently address HIV and OUD using various evidence-based strategies to improve outcomes among PWID [6, 7]. 

Several evidence-based interventions have been shown to reduce HIV acquisition, fatal and non-fatal opioid overdose, and other complications of injection drug use among PWID [8]. For example, HIV pre-exposure prophylaxis (PrEP) is recommended by the Centers for Disease Control and Prevention and the US Preventive Services Task Force for people who share injection equipment [8, 9]. The increased accessibility of HIV screening technology, including HIV self-testing (HIVST), has the potential to increase HIV testing rates and offers promise for engaging PWID in HIV treatment and prevention services, including PrEP [10–12]. Research findings demonstrate that HIVST is effective, convenient, and accurate in diagnosing HIV infection [10–12]. Similarly, medications for opioid use disorder (MOUD), such as buprenorphine and methadone, are evidence-based treatments for OUD that reduce non-prescribed opioid use, fatal and non-fatal overdose, HIV risk behaviors and, in the case of methadone, HIV incidence [13–15]. 

Despite compelling evidence, PrEP, MOUD, and HIVST remain underutilized in real-world settings, especially among PWID [7, 11]. A 2018 study using National HIV Behavioral Surveillance data showed that although 92% of PWID in Boston, Massachusetts had an indication for PrEP, only 2% reported past-year PrEP use [16]. Low PrEP and MOUD uptake among PWID has been attributed to limited access to medication information and prescribers, stigma, care fragmentation, competing priorities, and cost [7, 17–19]. HIVST remains underutilized due to the misconception that it is significantly less accurate than traditional laboratory testing. The sensitivity, or the ability of the test to correctly identify individuals with HIV, for HIVST is 92% compared to 99.7% for fourth generation antigen/antibody assays [20, 21]. Cost presents another barrier to HIVST uptake, as tests available over-the-counter are typically priced at approximately $40 [22, 23]. Given these social and structural barriers to HIVST, integrated and participant-centered interventions are needed to increase the uptake of these evidence-based measures among PWID.

Through foundational studies including qualitative interviews of PWID, our team demonstrated that active substance use was an important barrier to linkage to care [24]. We also elicited solutions proposed by participants, such as integrating PrEP and peer recovery coaching into substance use treatment programs [25]. We then pilot tested a peer recovery coaching intervention (CHORUS; Comprehensive HIV, Hepatitis C, and Opioid use disorder Response to the Unaddressed Syndemic) to increase PrEP and MOUD uptake at a low-barrier substance use disorder (SUD) bridge clinic. Previous literature highlighted that bridge clinics were accepted by patients and effective at providing medical services to those in need [26]. In another study, we evaluated and demonstrated the acceptability and feasibility of HIVST for persons who use drugs [6, 11]. 

In the current study, we will use a randomized control trial (RCT) to evaluate the efficacy of a peer recovery coaching intervention with baseline HIVST (CHORUS+) to increase initiation and adherence to PrEP among persons who inject opioids (PWIO). CHORUS + is a comprehensive, smartphone-supported intervention that integrates peer recovery coaching to address both HIV prevention and opioid treatment needs. The approach is guided by the Information-Motivation-Behavioral (IMB) Skills theoretical model to address known structural barriers to care engagement among this population [27]. The IMB model posits that behavioral change requires individuals to have accurate *information*, *motivation* and *behavioral skills* in the form of self-efficacy [27, 28]. We expand upon the IMB model to address known structural barriers to care engagement among this population [29]. 

Our primary hypothesis is that this participant-centered intervention will effectively promote PrEP initiation and adherence. Our secondary hypothesis is that the CHORUS + intervention will also improve MOUD initiation and adherence. This paper details the CHORUS + protocol and design, adhering to the Standard Protocol Items: Recommendations for Interventional Trials (SPIRIT) Statement [30]. 

## Methods: participants, interventions, and outcomes

### Study design

This two-arm RCT will enroll 284 PWIO who are negative for HIV at two sites. The first site, Boston Medical Center’s (BMC) Faster Paths to Treatment (FP), is a walk-in, on-demand, low-barrier substance use treatment bridge clinic. The second site, [Victory Programs, Inc. (VPI)], is a non-profit organization offering low-barrier and low-threshold services to the community [26, 31]. Both sites are located in a priority area for HIV prevention identified by federal agencies as part of the Ending the HIV Epidemic initiative [32]. 

**Fig. 1:**
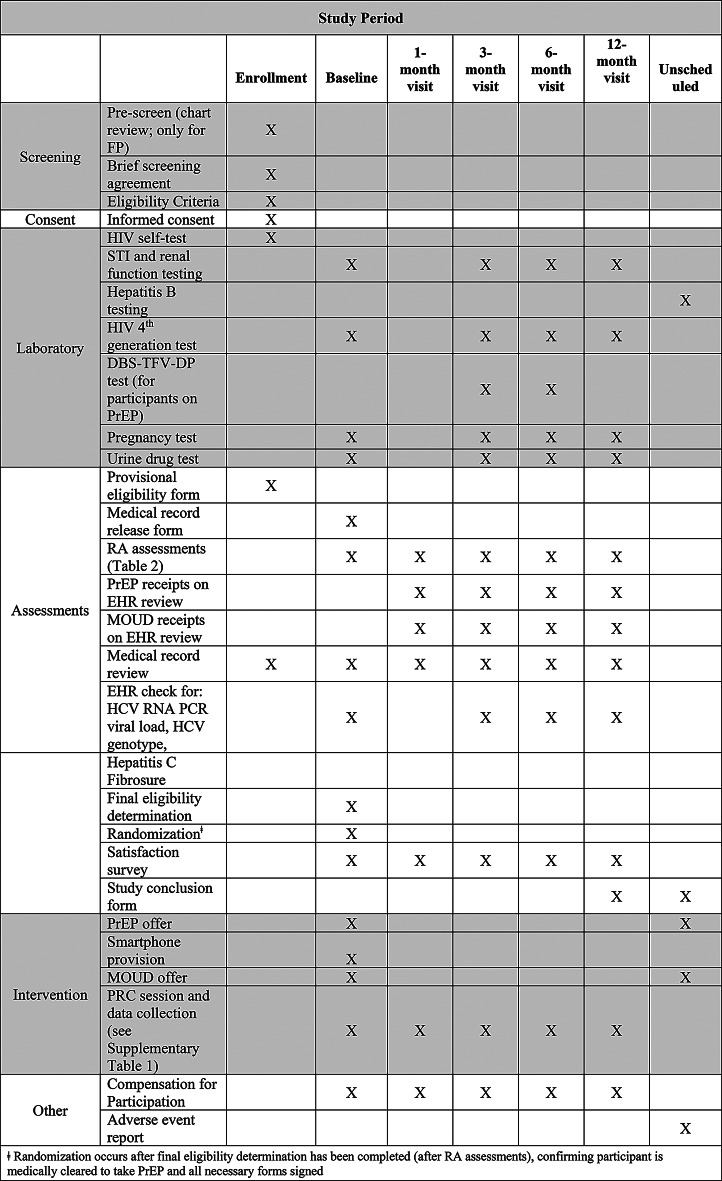
SPIRIT figure for the CHORUS + intervention

We will randomize consenting and eligible participants to either the CHORUS + intervention or the control (standard of care) arm (Fig. 1). We will provide each participant in the intervention arm with a study smartphone with an unlimited data, voice, and text plan to facilitate communication with their peer recovery coach (PRC) and improve retention. Smartphones will not include any special tools other than a secure, HIPAA-compliant videoconferencing application [33, 34]. The PRCs will implement the CHORUS + intervention. PRCs will undergo a 3-month training period covering motivational interviewing (MI) and the study protocol procedures. They will also receive annual booster training to maintain competency. PRCs will conduct MI sessions at baseline and throughout the 6-month intervention. In addition, sessions will also include modules from ‘Life-Steps for PrEP’ that have been modified to also address adherence to MOUD. Life-Steps sessions include 4 weekly and 2 monthly booster sessions. Additional details are provided in the description of the CHORUS + intervention and in Supplementary Table 1. PRCs will support participants in engaging and linking to PrEP and MOUD, and other necessary care including mental health and community resources to address social determinants of health (SDOH) such as housing and employment [35–37]. Participants who initiate PrEP and/or MOUD will also receive the modified Life-Steps intervention. To ensure adherence to the intervention content, we will audio-record all sessions between the coaches and participants. A randomly selected 10% of these recordings will be reviewed and rated for fidelity using standardized fidelity scales. Participants randomized to the control arm will be provided with contact information for community programs providing PrEP- and MOUD-related services. They will not be assigned to a PRC or offered PrEP or MOUD, nor will they undergo motivational interviewing or receive a study smartphone.

**Fig. 2:**
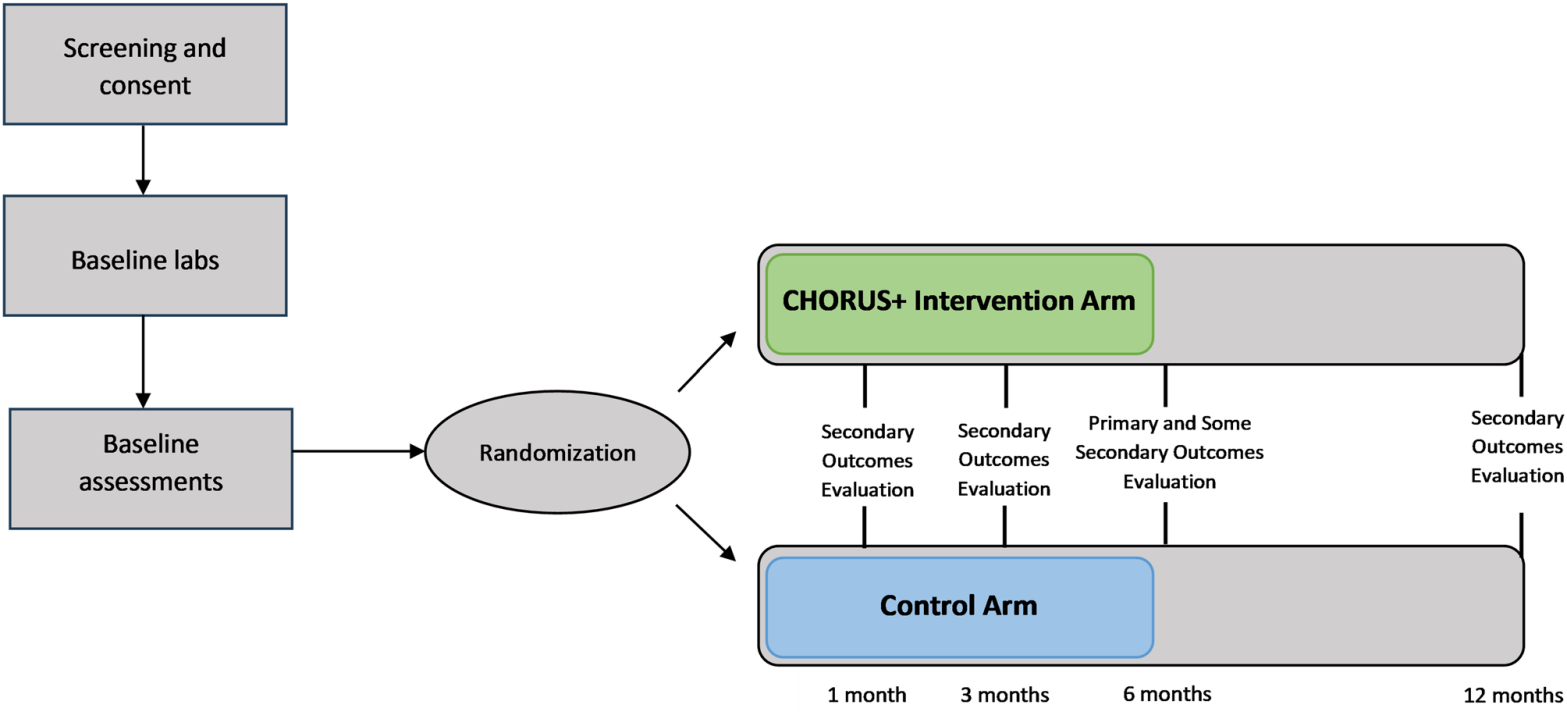
CHORUS+ (Comprehensive HIV and Opioid use disorder Response to the Unaddressed Syndemic) study design. *Intervention arm*: peer recovery coach, mobile phone, PrEP and MOUD offered; *Control arm*: information on local resources and places to access PrEP, MOUD, and harm reduction. Of note, the 1-month study visit does not include any laboratory testing

We will follow all participants in both study arms for 12 months, during which they will undergo RA-administered assessments at baseline, 1-, 3-, 6-, and 12-month study visits (Fig. 2). Each participant will be compensated for participating in the study. The BUMC IRB approved the study protocol (protocol # H-43487); the following description of the protocol is based on Version 1.18, most recently updated on 6/26/25.

### Inclusion and exclusion criteria

Participant eligibility criteria are outlined in Table 1. In brief, participants must be 18 years or older, speak English, self-report injecting opioids in the past 6 months, and be medically cleared to take PrEP and MOUD (normal renal and other PrEP-related laboratory studies). Individuals will be excluded if they have been diagnosed with HIV previously, are pregnant at baseline, are already enrolled in another intervention study, or express desire to hurt themselves or others.

**Table 1:** Inclusion and exclusion criteria for CHORUS+

Inclusion	Exclusion
• 18 years or older • English speaking • Injected opioids within the past 6 months • Willing to provide contact information for two family members or friends • Willing to sign medical record release form • Medically cleared to take PrEP and MOUD (normal renal and other labs)^ǂ^	• Persons living with HIV • Persons who express desire to harm themselves or others • People currently in another intervention study • Pregnant people*

*Only excluding pregnant people at baseline; people who become pregnant during the study will not be disenrolled

ǂIncludes HIV and creatinine testing

Note that participants who have already initiated PrEP or MOUD before enrollment are still eligible for the study

### Recruitment and pre-screening

Participant recruitment protocols will vary by study site based on available pre-screen information. When possible, we will first assess pre-screen eligibility criteria, as approved by the IRB, by reviewing participants’ HIV status and injection drug use history using both electronic health records (EHR) and self-report. At the FP site, the RA will review participants’ EHR prior to scheduled visits to determine potential study eligibility based on opioid injection history and HIV status. The RA will inform clinicians of potentially eligible participants. During the patient’s clinical visit, the FP’s site clinician will briefly introduce the study to the patients. If patients are interested in participating, the FP clinical staff will inform RAs by communicating either in-person, via phone, through secure BMC email, or EHR secure chat. They will also facilitate a warm handoff to the RA after the clinical visit.

For recruitment at the VPI site, fliers will advertise the study. The VPI site staff and the RA will inform individuals visiting the center that there is an opportunity to participate in a study including peer recovery coaching focused on HIV prevention and OUD treatment. Individuals who are interested in knowing more will be screened for eligibility.

### Eligibility screening and informed consent

The research team will assess participant inclusion and exclusion criteria through self-report and completion of a pregnancy test (when relevant) for individuals who meet pre-screen eligibility criteria. Potential participants will undergo the informed consent process, during which they will be encouraged to ask questions. The RA will emphasize that participation in the study is voluntary and will not impact the care or services they receive at the FP or VPI sites. If an individual agrees to participate, the RA will collect their electronic signature using REDCap, an electronic data capture tool.

### Randomization and blinding

Following assessments and laboratory testing, the participant’s study inclusion and exclusion criteria (Table 1) will be formally assessed using a case report form (CRF), which includes HIVST, laboratory testing for renal function and pregnancy. Participants will be randomized in a 1:1 ratio to either the CHORUS + intervention arm (*n* = 142) or the control arm (*n* = 142). We will stratify randomization by recruitment site (BMC or Victory Programs), sex assigned at birth (female or male) or race (Black or other) using randomly varying block sizes of 4 and 6. Participant allocation will be concealed from study staff to ensure that the allocation sequence is not accessible to those interacting with participants until assignment occurs. Investigators involved in data analysis, but not in the study’s conduct, will remain blinded to treatment group assignments. We will maintain semi-blind conditions, with analyses by treatment group deferred until study masking is formally broken according to the statistical analysis plan (SAP).

### Timing of assessments

The RA will administer assessments at baseline (after the participant is consented and enrolled into the study) and during follow-up visits at 1-, 3-, 6-, and 12-month visits post enrollment (Fig. 1).

All RAs will be trained in the ethical conduct of human subjects research, in the proper administration of study-related procedures, and CRF completion. This will help mitigate bias and ensure that the intention of each question is clearly conveyed and recorded. RAs will contact participants who have agreed to receive reminders about their upcoming appointments prior to 1-, 3-, 6-, and 12-month assessments.

### The CHORUS + intervention

CHORUS + is a novel, theory-based, smartphone-supported intervention using peer recovery coaching to implement a comprehensive approach to the syndemic of OUD and HIV. PRCs meet individually with participants, either in-person or using their study phones, with the aim of motivating them to engage in PrEP and OUD care, by supporting the recognition of their own strengths to make positive changes in their lives and ultimately improve HIV prevention and OUD outcomes.

The Information-Motivation-Behavioral (IMB) Skills theoretical model informed our intervention design [29]. Within this framework, PRCs will offer information about PrEP and MOUD and use motivational interviewing to assist participants randomized to the intervention in setting goals related to PrEP and MOUD adherence. PRCs will encourage participants to set their own goals, identify their strengths, learn behavioral skills, and help them navigate their desired outcomes (Supplementary Fig. 1). The PRCs will also help individuals address and navigate structural barriers such as employment, housing, and access to healthcare as it may hinder initiation and adherence to PrEP and/or MOUD.

We will organize the intervention into 2 phases over 6 months: (1) initiation phase at enrollment (pre-initiation phase), and (2) maintenance phase (post-initiation phase) (Supplementary Table 1).

#### Initiation phase

The PRCs will perform MI, discuss substance use and MOUD. PRCs will help participants identify and use their strengths to achieve personal recovery goals. In addition to eliciting and enhancing participants’ strengths, PRCs will work directly with individuals to develop and implement self-directed recovery wellness plans, which will include attention to HIV prevention, and offer resources to operationalize those plans [29]. The PRCs will also use motivation engagement techniques and help participants arrange and keep appointments with their clinicians to discuss PrEP and MOUD. After the initial MI session, follow-up sessions will focus on PrEP and MOUD initiation, challenges related to SDOH that affect PrEP and MOUD maintenance, as well as assistance with accessing resources such as housing and employment opportunities [29]. Participants will use their study smartphone for ease of communication with the PRC and may choose to meet with the PRCs either in-person or over the phone.

#### Maintenance phase

We will integrate modules from the ‘Life-Steps for PrEP’ evidence-informed intervention to assist with adherence whenever participants decide to initiate PrEP and/or MOUD [38]. ‘Life-Steps for PrEP’ is an individual, facilitator-led intervention with 4 weekly and 2 monthly booster sessions, as outlined in Supplementary Table 1 [38]. These sessions will assist participants with setting PrEP and MOUD-related adherence goals and develop plans for continued use, when applicable, even after completion of the intervention. We adapted Life-Steps modules to better address injection drug use and MOUD (Supplementary Table 1). The PRCs will review steps involved with using medication and developing a back-up plan. In subsequent sessions, the PRCs will assist with more intensive problem-solving techniques as needed, and our research protocols will be adapted for consistency in managing issues across participants.

### Measures

At baseline, 1-, 3-, 6-, and 12-months, participants will complete RA-administered assessments for HIV risk perception, PrEP and MOUD knowledge and use, substance use, sexual behaviors, depression, anxiety, barriers to medical care and SDOH (Fig. 1). Demographic information such as race/ethnicity and age will only be collected at baseline. The study team will conduct medical record reviews throughout the study. Biological measures including laboratory-based HIV 4th generation Ag/Ab blood tests, standard of care tests, and substance use urine toxicology tests for all participants regardless of randomization at 3-, 6-, and 12-months. We will also perform dried blood spot tenofovir-diphosphate (DBS TFV-DP) tests for all individuals who report taking PrEP at 3-, and 6-months. We will assess PrEP and/or MOUD adherence as noted below including biomarkers, active prescription refills in participants’ medical records, and by self-report.

The primary outcome is that participants in the CHORUS + intervention arm will demonstrate greater adherence to PrEP compared to participants in the control arm, as measured by DBS-TFV-DP level at 6 months post-enrollment. Secondary outcomes will assess adherence to PrEP at 1-, 3-, and 12-months post enrollment, and MOUD at 1-, 3-, 6- and 12-months post-enrollment, evaluating whether the intervention arm demonstrates improved adherence to medications as compared to the control arm at each timepoint compared to baseline. We will measure mediators of intervention effects by self-report through RA-administered assessments at each study visit. These include HIV knowledge and risk perception, PrEP/MOUD perception, motivation, and knowledge.

**Table 2:** Assessments administered throughout the CHORUS + intervention

Items to Complete	Study Time Point						
	Screening	Baseline	1-Month	3-Month	6-Month	12-Month	As Needed
Forms							
Pre-Screen Agreement	X						
Pre-Screener for eligibility	X						
Eligibility screener	X						
Consent form		X					
Disposition form		X	X	X	X	X	
Randomization case report form^ǂ^		X					
Adverse event form							X
Protocol deviation form							X
**Assessments**							
Assessments*		X	X	X	X	X	
Medical history form [41]		X					
*List of Assessments Administered							
Demographics		X					
HIV and Hepatitis C testing history [42]		X	X	X	X	X	
PrEP and MOUD use and adherence [43]		X	X	X	X	X	
Perceived risk of HIV infection [44]		X	X	X	X	X	
PrEP attitudes, stigma, and intentions [28, 45]		X	X	X	X	X	
Constructs for PrEP-related IMB Skills [46]		X	X	X	X	X	
DSM-5 Opioid Use Disorder [47]		X	X	X	X	X	
Drug use: ASI [48]		X	X	X	X	X	
Risk behavior survey: drug injection [49]		X	X	X	X	X	
Alcohol use: AUDIT [50]		X	X	X	X	X	
Risk behavior survey: sexual behaviors [49]		X	X	X	X	X	
Depressive symptoms (PHQ-8) [51]		X	X	X	X	X	
Anxiety (GAD-7) [52]		X	X	X	X	X	
Social support scale [53]		X	X	X	X	X	
Social determinants of health [54–66]		X	X	X	X	X	
Health care utilization [67]		X	X	X	X	X	
Barriers to medical care part 1 [68]		X	X	X	X	X	
Barriers to medical care part 2 [68]		X	X	X	X	X	
Satisfaction survey		X	X	X	X	X	

ǂRandomization will occur after participants have been medically cleared to take PrEP

### Statistical analyses

The statistical analysis plan includes three components: (1) a primary analysis to evaluate the efficacy of the CHORUS + intervention on PrEP adherence at six months under a standard clinical trial framework; (2) secondary analyses to assess additional outcomes at six months and examine longitudinal trends in PrEP and MOUD secondary outcomes at 1-, 3-, 6- and 12- months; and (3) exploratory analyses to evaluate mechanisms of intervention effect using structural modeling approaches.

For the primary analysis, we will use an intent-to-treat (ITT) population, defined as all randomized participants who complete the baseline assessment. We will analyze participants according to their assigned treatment group, regardless of intervention adherence or continued study engagement. We will use logistic regression to model the binary adherence outcome, adjusting for the stratification variables used during randomization: site, sex assigned at birth, and race. If the number of PRCs is limited, PRC will be included as a fixed effect rather than a random effect to adjust for between-coach differences while avoiding instability in variance estimation associated with a small number of clusters. We will address missing outcome data using multiple imputations under a missing-at-random assumption to reduce bias and improve precision. We will perform sensitivity analyses including per-protocol (PP) comparisons based on adherence to the intervention. We will report results as odds ratios with 95% confidence intervals, and we will assess statistical significance using a two-sided alpha of 0.05. This analysis will serve as the formal test of intervention efficacy. We will analyze secondary PrEP and MOUD outcomes at 6 months using the same approach. We will not perform any adjustments for multiple testing. We will finalize a detailed statistical analysis plan that will be approved by the study team prior to unblinding to ensure analytical integrity.

We will examine additional secondary analyses of PrEP and MOUD adherence at 1-, 3-, 6- and 12- months, using multilevel growth curve modeling. The models will include random intercepts and slopes to capture individual trajectories, and we will adjust stratification variables and PRCs. We will evaluate differences in slopes by treatment group. While these outcomes are prespecified, we will interpret the associated analyses as supportive and intended to assess consistency of intervention effects over time and across domains.

We will also investigate exploratory analyses by evaluating the intervention’s potential mechanisms. We will use multilevel structural equation modeling to examine hypothesized mediators such as HIV risk perception, PrEP and MOUD knowledge, and motivation. These models will estimate both direct and indirect effects of the intervention on adherence outcomes. We will interpret mediation analyses descriptively as they are intended to inform future refinement of intervention components. We will evaluate model fit using standard diagnostics such as residual plots, Bayesian Information Criterion, and fit indices for structural models where applicable.

We will base conclusions from analyses on both the statistical significance of parameters and the magnitude of observed effect sizes. We will conduct all analyses using SAS version 9.4.

### Interim analyses and data monitoring

No interim efficacy analyses or formal stopping rules are planned, given the study’s minimal risk classification. The Data and Safety Monitoring Board will review study progress and safety outcomes every six months. Annual reports will also be submitted to the institutional review board and the study sponsor.

### Sample size

The CHORUS pilot data indicated that among those who completed the intervention, 43% were on PrEP at the end of the study compared to less than 1% in the control group from the literature [6, 39]. Although these findings are encouraging, these findings are based on a different outcome and smaller sample size. Therefore, for the current study, the primary outcome is adherence to PrEP based on laboratory marker results at 6 months post-enrollment. We conservatively estimate that 12% of participants in the CHORUS + intervention will meet the adherence criteria for PrEP at 6 months. Based on real-world reports among PWID, we anticipate 2% of participants in the control arm will meet PrEP adherence criteria [16]. These assumptions yield a small to moderate effect size of 0.4. By enrolling 142 participants in each arm using a Pearson chi-square test for two independent proportions, this study will have 91.5% power to detect a difference in the primary outcome of 12% adherence to PrEP in the intervention arm as compared to 2% in the control arm, with a two-sided type I error rate of 5%. We anticipate 25% non-differential loss to follow up at 6 months, yielding an evaluable sample size of 214 participants for the primary outcome, which ensures 82.2% power, under the same assumptions. We will have at least 80% power for the proposed secondary outcomes.

### Data collection, management, and confidentiality

The study team will conduct all interviews and interventions in private locations at each site. Each study participant will be assigned a unique study identifier. Over the duration of the study, the study team will collect all data (i.e., CRFs, specimens) from all participants and the PRCs will electronically record all interactions with participants (in-person, telephone, text, or videoconference) directly into a web-based mobile site. The data management team will develop and validate a REDCap data entry application to store all participant data.

### Dissemination plan

We will disseminate study findings at national conferences, meetings with key informants and community partners, and publications in scholarly journals.

## Discussion

The rise in human immunodeficiency virus (HIV) cases among PWID during the overdose crisis underscores the need to expand upon the prevention methods such as PrEP [1, 3]. We followed sequential steps to develop CHORUS+ (Comprehensive HIV and OUD Response to the Unaddressed Syndemic), a comprehensive HIV prevention and OUD treatment intervention.

Our initial formative study showed that active substance use was an important barrier to linkage to care [6]. We then conducted in-depth interviews of individuals with substance use disorder and identified solutions proposed by participants, including integration of HIV prevention in the form of PrEP within substance use treatment programs, and the provision of support services with peer recovery coaching [6]. Next, we developed and pilot tested the CHORUS intervention to determine feasibility, acceptability and potential for efficacy of a 6-month peer recovery coaching intervention [6]. Data showed that PrEP initiation occurred late in the intervention (at approximately 3 months), in part due to delays related to HIV testing, delivery of results, and evaluation for PrEP [6]. 

Informed by these data, we enhanced the CHORUS intervention (now called CHORUS+) by providing HIV self-testing (HIVST) to potentially accelerate discussion about HIV prevention and PrEP; this modification was feasible and acceptable at a low-barrier community-based organization serving people who use drugs (PWUD) in our formative work [11]. In addition, all participants noted that they would recommend this form of HIV testing to others. As part of the current randomized controlled trial, modifications to the CHORUS intervention such as HIVST at recruitment, are provided along with elements of the original intervention including a 6‑month peer recovery coaching intervention to promote engagement with and sustained use of PrEP and MOUD. HIVST at recruitment enables participants to quickly learn their HIV status. This approach allows for early discussions about PrEP and MOUD initiation as the conversation will be informed by objective data on negative HIV testing results.

To test the efficacy of the CHORUS + intervention, we will collaborate with a substance use bridge clinic as well as a low-barrier, low-threshold community-based organization, both of which are utilized by PWIO. Previous literature has evaluated the importance of PrEP initiation and adherence to mitigate HIV transmission among PWIO; partnership with low-barrier community-based organizations and the provision of evidence-based measures (PrEP, MOUD, and HIVST) using a comprehensive approach represent promising innovations warranting formal evaluation within an RCT design [40]. The data from CHORUS + will inform future actions towards integrated care through a pragmatic clinical trial assessing the impact of CHORUS + in standard care circumstances. These data could propel the field by reshaping the existing fragmented method of addressing OUD and its infectious complications, such as HIV (Table 2).

## Conclusion

Informed by formative work and in partnerships with low-barrier, low-threshold organizations, we followed sequential steps to develop a theory-based comprehensive intervention to increase PrEP adherence for PWIO. Our approach is innovative, as it leverages low-barrier models that meet the needs of PWIO in an on-demand technology-supported capacity; it provides PrEP and MOUD focused peer support in areas traditionally focused on just MOUD; it leverages findings from the CHORUS study’s formative work to accelerate PrEP initiation when people seek care in low-barrier settings; it integrates Life-Steps for PrEP and MOUD into PRC sessions to support medication adherence; it addresses SDOH such as access to healthcare; and it promotes rapid PrEP uptake by offering HIVST to all participants at recruitment, as participants quickly learn their HIV status, allowing discussions about PrEP and MOUD initiation.

## Supplementary information

Below is the link to the electronic supplementary material.

Supplementary Material 1

## Data Availability

No datasets were generated or analysed during the current study.

## Abbreviations

HIV: Human Immunodeficiency Virus
PrEP: Pre-exposure prophylaxis
MOUD: Medications for opioid use disorder
OUD: Opioid use disorder
PWIO: Persons who inject opioids
IRB: Institutional review board
MI: Motivational interviewing
RCT: Randomized controlled trial
DBS: Dried blood spot
DBS-TFV-DP: Dried blood spot tenofovir-diphosphate
HIVST: HIV self-test
FP: Faster Paths clinic
VPI: Victory Programs, Inc
PRC: Peer recovery coach
RA: Research assistant
BMC: Boston Medical Center
DSMB: Data safety monitoring board
SUD: Substance use disorder
PWUD: People who use drugs
