# Supplementary material for: Pre-exposure prophylaxis (PrEP) and medications for opioid use disorder for persons who inject drugs: the CHORUS + randomized controlled trial study protocol
Source: Addict Sci Clin Pract. 2025 Dec 25;21:17. doi: 10.1186/s13722-025-00634-2 (PMC12849554; doi:10.1186/s13722-025-00634-2)

**Supplementation Table 1.** Phases of peer recovery coaching sessions

| Phase | Timeline | Session | Focus |
| --- | --- | --- | --- |
| Initiation Phase | At enrollment (or within 1 month) | Initial MI session | - Baseline interview |
|  | PRE-INITIATION | | |
|  | 1 week post enrollment | Pre-initiation session 1 | - Exploration of goals and needs related to HIV prevention and substance use, connection with resources |
|  | 2 weeks post enrollment | Pre-initiation session 2 | - Exploration of goals and needs related to HIV prevention and substance use, connection with resources |
|  | 3 weeks post enrollment | Pre-initiation session 3 | - Exploration of goals and needs related to HIV prevention and substance use, connection with resources |
|  | 4 weeks post enrollment | Pre-initiation session 4 | - Exploration of goals and needs related to HIV prevention and substance use, connection with resources |
|  | 6 weeks post enrollment | Pre-initiation session 5 | - Connection with resources |
|  | 8 weeks post enrollment | Pre-initiation session 6 | - Connection with resources |
|  | 12 weeks post enrollment | Pre-initiation session 7 | - Motivating engagement in PrEP and OUD treatment |
|  | 16 weeks post enrollment | Pre-initiation session 8 | - Motivating engagement in PrEP and OUD treatment |
|  | 20 weeks post enrollment | Pre-initiation session 9 | - Sustainability |
|  | 24 weeks post enrollment | Pre-initiation session 10 | - Sustainability |
| POST-INITIATION* | | | |
| Maintenance Phase | 1 week post initiation | Life-Steps session 1 | - Education, motivational exercise, dosing schedule |
|  | 2 weeks post initiation | Life-Steps session 2 | - Check-in, problem solving |
|  | 3 weeks post initiation | Life-Steps session 3 | - Check-in, sexual risk behavior education, problem-solving |
|  | 4 weeks post initiation | Life-Steps session 4 | - Check-in, problem-solving, planning for future |
|  | 6 weeks post initiation | General check-in | - Connection with resources - Follow-up on adherence - Problem-solving |
|  | 8 weeks post initiation | Life-Steps booster session 1 | - Check-in, problem-solving, planning for future |
|  | 10 weeks post-initiation | General check-in | - Connection with resources - Follow-up on adherence - Problem-solving |
|  | 12 weeks post initiation | Life-Steps booster session 2 | - Check-in, problem-solving, planning for future |
|  | 16 weeks post initiation | Life-Steps post counseling | - Connection with resources - Follow-up on adherence - Problem-solving - Sustainability |
|  | 20 weeks post initiation | Life-Steps post counseling | - Connection with resources - Follow-up on adherence - Problem-solving - Sustainability |
|  | 24 weeks post initiation | Life-Steps post counseling | - Connection with resources - Follow-up on adherence - Problem-solving - Sustainability |
| Note: participants will work with PRCs for 6 months after enrollment. At any point, a participant can choose to initiate PrEP or MOUD. Once a participant has initiated either medication, they will transition to the post-initiation schedule and continue that schedule until the end of the 6-month intervention period. | | | |
| Abbreviations: MI=motivational interviewing; PrEP=pre-exposure prophylaxis; MOUD=medications for opioid use disorder; HIV=human immunodeficiency virus | | | |

***Supplementary Figure 1*.** Framework applied to pre-exposure prophylaxis (PrEP) and medications for opioid use disorder (MOUD)


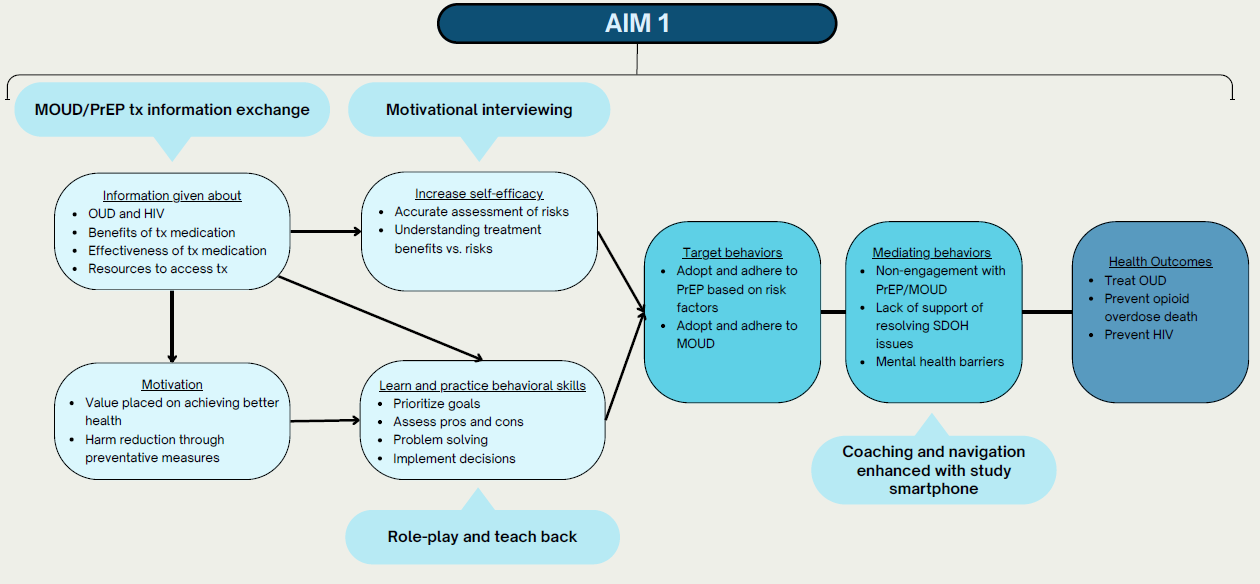

Supplement: Supplementary file 1 — Supplementary Material 1 [file 13722_2025_634_MOESM1_ESM.docx]
